# Temporal Evaluation of the Surface Area of Treated Skin Ulcers Caused by Cutaneous Leishmaniasis and Relation with Optical Parameters in an Animal Model: A Proof of Concept

**DOI:** 10.3390/s23135861

**Published:** 2023-06-24

**Authors:** Sergio Londoño, Carolina Viloria, Sandra Pérez-Buitrago, Javier Murillo, Deivid Botina, Artur Zarzycki, Johnson Garzón, Maria C. Torres-Madronero, Sara M. Robledo, Franck Marzani, Sylvie Treuillet, Benjamin Castaneda, July Galeano

**Affiliations:** 1 Grupo de Investigación e Innovación Biomédica, Instituto Tecnológico Metropolitano, Medellín 050034, Colombia; 2 Grupo de Investigación en Dispositivos Médicos, Departamento de Ingeniería, Pontificia Universidad Católica del Perú, Lima 15088, Peru; 3 Programa de Estudio y Control de Enfermedades Tropicales—PECET, Facultad de Medicina, Universidad de Antioquia, Medellín 050010, Colombia; 4 Laboratoire ImViA, Université de Bourgogne, BP 47870, 21078 Dijon Cedex, France; 5 Independent Researcher, Envigado 055422, Colombia; 6 Grupo de Óptica y Espectroscopía, Universidad Pontificia Bolivariana, Medellín 050031, Colombia; 7 Research Group on Smart Machine and Pattern Recognition, MIRP Laboratory, Instituto Tecnológico Metropolitano, Medellín 050013, Colombia; 8 Laboratoire Pluridisciplinaire de Recherche Ingénierie des Systèmes, Mécanique, Énergétique—PRISME, Université d’Orléans, 45072 Orléans, France; 9 Department of Biomedical Engineering, University of Rochester, Rochester, NY 14620, USA; 10 Grupo de Investigación Materiales Avanzados y Energía MatyEr, Instituto Tecnológico Metropolitano, Medellín 050013, Colombia

**Keywords:** cutaneous leishmaniasis, 3D modeling, skin ulcer, optical parameters, follow-up

## Abstract

Cutaneous leishmaniasis (CL) is a neglected disease caused by an intracellular parasite of the *Leishmania* genus. CL lacks tools that allow its understanding and treatment follow-up. This article presents the use of metrical and optical tools for the analysis of the temporal evolution of treated skin ulcers caused by CL in an animal model. *Leishmania braziliensis* and *L. panamensis* were experimentally inoculated in golden hamsters, which were treated with experimental and commercial drugs. The temporal evolution was monitored by means of ulcers’ surface areas, as well as absorption and scattering optical parameters. Ulcers’ surface areas were obtained via photogrammetry, which is a procedure that allowed for 3D modeling of the ulcer using specialized software. Optical parameters were obtained from a spectroscopy study, representing the cutaneous tissue’s biological components. A one-way ANOVA analysis was conducted to identify relationships between both the ulcers’ areas and optical parameters. As a result, ulcers’ surface areas were found to be related to the following optical parameters: epidermis thickness, collagen, keratinocytes, volume-fraction of blood, and oxygen saturation. This study is a proof of concept that shows that optical parameters could be associated with metrical ones, giving a more reliable concept during the assessment of a skin ulcer’s healing.

## 1. Introduction

Leishmaniasis is a disease caused by an intracellular parasite of the *Leishmania* genus. The World Health Organization declared it a public health problem. It is present in 98 countries, with the highest incidence in tropical and endemic areas of the Western Hemisphere [1]. Approximately 12 million people worldwide suffer from leishmaniasis, and two million cases are reported each year [2]. Leishmaniasis affects both skin and mucous membranes. Cutaneous leishmaniasis (CL) generates ulcers that regularly appear at the site of sandfly vector stings.

In cutaneous leishmaniasis, the response to treatment is defined according to the clinical monitoring of the appearance of lesions after the end of treatment in terms of cure, failure, and relapse, where cure corresponds to complete re-epithelialization with the complete absence of inflammatory signs or erythema in all lesions; therapeutic failure is defined as the absence of complete re-epithelialization or the presence of an infiltrate and erythema; and relapse is defined as the healing of lesions by week 12 but the reappearance of lesions after that initial cure [3,4,5]. The manual measurement of the lesion area and visual inspection are standard analyses made by physicians to determine skin ulcer evolution. This is known as the manual ruler method, which is suggested in medical wound care guidelines and specialized communities [6,7]. Since these are observer-dependent analyses, it is necessary to develop new control strategies that allow these parameters to be obtained more precisely and with a greater degree of effectiveness [8] to facilitate data interpretation of the evolution over time and identify treatment effectiveness.

Three-dimensional (3D) modeling is a tool that can be used to analyze the temporal evolution of ulcer healing [9,10,11,12]. Three-dimensional modeling can be based on the photogrammetry approach [13,14], in which the representative points of an object are joined in the form of elements with a geometric surface. It is achieved by combining several points of view by a sequence of phases, such as interest points’ detection, correspondence, and the reconstruction of points. Three-dimensional models can provide accurate surface measurements on curved areas.

Additionally, 3D modeling based on conventional cameras represents a low-cost, fast, and easy-to-implement method since it could be applied in difficult patient-care areas such as rural points of care or basic-level consulting rooms. This is the case with CL, which is present mainly in low-income rural areas. Therefore, the equipment to monitor the healing of the ulcer is expected to be easy to transport and is expected to work in harsh environmental conditions (high humidity and high temperature) [15].

On the other hand, the composition of the tissues denotes optical properties that allow interaction with irradiated light. Color and multispectral imaging can be used for wound healing evaluation [16,17]. For the estimation of biological variables in the tissues, diffuse reflectance models are used which allow the quantification of the biological components of the skin through a spectral signature [18,19]. Spectral analysis is widely used to analyze cutaneous pathologies by quantifying parameters such as epidermis thickness, keratinocytes, collagen, oxygen saturation (SpO_2_), etc. [20,21].

Both 3D-surface reconstruction and optical properties could allow for better understanding and follow-up of the illness. This could avoid unnecessary dosages of medication being provided to the patient, reducing possible side effects [15].

To analyze the utility of vision systems in CL treatment follow-up, this article proposes a combination of 3D reconstructions and diffuse reflectance optical measurements for the follow-up of treated skin ulcers caused by CL in an animal model. As a methodology, the measurements were taken from the injured areas during three temporal stages of the disease: the beginning of the infection process, during, and after the treatment. After correlating the ulcers’ surface areas obtained from the 3D study with the optical parameters, it is possible to observe that the disease’s evolution depends on each individual’s physiological conditions, immune response, and type of scarring. These results show that it is possible to improve the understanding of the disease’s evolution and propose three-dimensional analysis, together with optical ones, to assist physicians in monitoring CL healing.

## 2. Materials and Methods

### 2.1. Animals

Golden hamsters (*Mesocricetus auratus*), which were inbred, both sexes, six weeks old, and weighing approximately 150 g, raised under specific-pathogen-free (spf) conditions in the animal facility of the University of Antioquia and housed in controlled environmental conditions, were experimentally infected with 1 × 108 promastigotes in stationary-growth-phase *Leishmania braziliensis* (MHOM/CO/88/UA301) (*n* = 8) or *L. panamensis* (MHOM/CO/87/UA140) (*n* = 8), in 100 μL of PBS, intradermally on the skin of the back (1 inch above the base of the tail). Six to eight weeks after the experimental infection, when the hamsters developed an ulcer of a minimum size of 4 × 4 mm^2^, the hamsters inoculated with each *Leishmania* species were randomly distributed into two groups. In the group inoculated with *L. panamensis*, 4 hamsters were treated with 40 mg/day of a mixture of glycolic and ethanolic extracts (1:1) of *Caesalpinia spinosa*, administered topically (LPET); and 4 hamsters were treated with 200 µL (1 µg/µL) of conventional meglumine antimoniate, every two days, intralesionally (LPCT). Each of the treatments lasted 30 days. In the group inoculated with *L. braziliensis*, 4 hamsters were treated with the extract mixture (LBET), and 4 were treated with meglumine antimoniate (LBCT).

To evaluate the response to treatment, once it had finished, a follow-up was carried out every 15 days for three months that consisted of weight control and the measurement of the ulcer. This follow-up was carried out to determine if there was any improvement (a reduction in the ulcer compared to the mean at the start of the treatment), a cure (a scar where there was an ulcer), or failure (ulcer remaining the same or larger compared to the start of the treatment measure). The welfare of the animals was monitored daily during the study to detect changes in behavior, food and water consumption, and the appearance of urine and feces.

The start of the treatment was the first date for data acquisition, the second date of measurements was at the end of the treatment (day 30 of treatment), and subsequent measurements were every 15 days for three months, corresponding to the post-treatment period. For the analysis presented in this article, only the hamsters that arrived at the end of the post-treatment period were considered.

For data acquisition, hamsters were anesthetized using a mixture of ketamine (50 mg/mL) and xylazine (20 mg/mL) in a ratio of 9:1, intraperitoneally [18]. Once the animals were anesthetized, an area of approximately 3 cm × 3 cm of fur on the dorsal skin was removed using a shaving device, avoiding irritation or laceration. Based on visual inspection, the clinical criterion of being cured was given by measurements of the injured area at the end of the study compared to those at the beginning of the treatment. When the elimination of the area with complete epithelialization was observed, the hamster was classified as cured, but when a partial reduction in the area or no reduction in the size of the lesion was observed, the hamsters were classified as improved or not cured, respectively [22]. 

This procedure was approved by the Ethics Committee for Animal Experimentation of the University of Antioquia (Minutes No. 110 of 17 May 2017), Colombia, and follows the national animal protection law [23].

### 2.2. Spatial Data Acquisition

The evolution of the disease was monitored by a video recording of the ulcer developed on each hamster. This was achieved using a Sony NEX-5T camera (Sony Corporation, 1-7-1 Konan Minato-ku Tokyo, 108-0075 Japan) with a 16-megapixel resolution and JPEG HD-compatible recording format. The video shots were acquired every two weeks during the treatment and post-treatment periods. A total of five dates of video records were obtained for each hamster.

**Figure 1:**
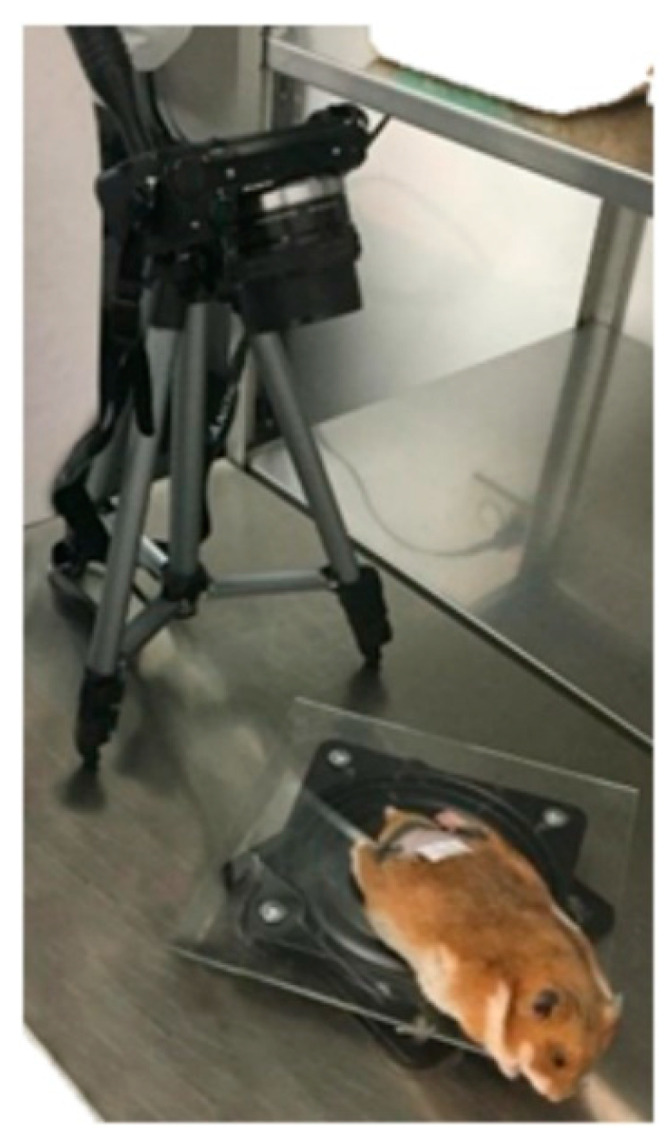
Experimental setup for the acquisition of video.

For a standard video acquisition process, a mobile platform was developed consisting of a rotating base on which the hamster was positioned (Figure 1). This platform allowed 360° rotations to completely visualize the ulcer from the camera lens. The camera was positioned at a 50 mm focal length on a tripod with an inclination angle of 80°, allowing for better visualization of the area of interest [8].

To record the video, each anesthetized hamster was placed in the middle of the turntable in the prone position and manually moved until two complete turns were achieved. The recorded videos included enough information to ensure a successful 3D reconstruction (at least 20 frames).

Three-dimensional models of CL ulcers were obtained using Agisoft Photoscan software, version 1.5.2.

### 2.3. Data Processing and 3D Reconstruction

**Figure 2:**
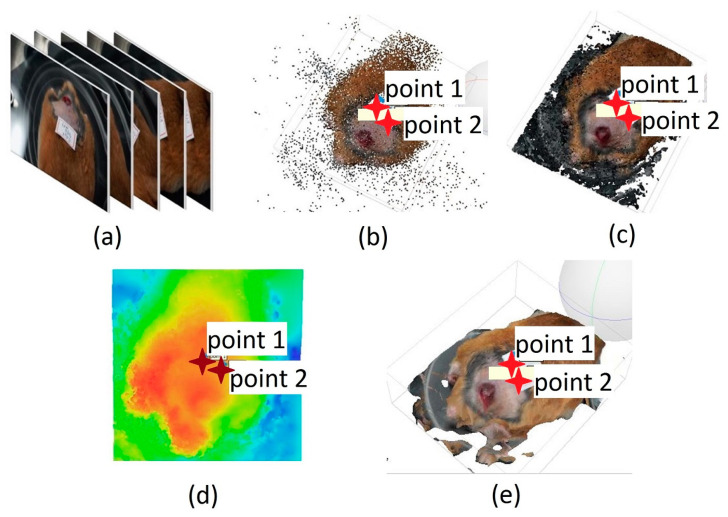
Three-dimensional model acquisition process. (**a**) Extracting frames from video recording and matching characteristics alignment. (**b**) Creation of the dense point cloud. (**c**) Generation of the 3D mesh. (**d**) DEM model. (**e**) Final result of the process.

The processing of the acquired images started by selecting 50 frames extracted from each video (Figure 2a). This number of frames was established as optimal to have better computational performance with the Agisoft PhotoScan software, based on the 3D reconstruction of multiple views from still images [24] in which the frames were loaded to obtain 3D reconstructions [25,26,27,28]. Reconstruction was carried out using a series of intuitive commands designed for this purpose. The first stage was the alignment of the images using a technique known as matching characteristics alignment (Figure 2b). This technique recovers the relative position and orientation of each image in a set. A dense point cloud was then created in which the image pixels were projected back to the original spatial locations with a high-quality setting to create the 3D mesh and give the respective texture to the reconstruction (Figure 2c). After obtaining the 3D model, its view was modified until a frontal arrangement of the ulcer was obtained. This was carried out to reduce the parallax error concerning the position of the camera, and from this view build the DEM model (Digital Model of Elevation) (Figure 2d) [8]. This model is a numerical data structure representing the spatial distribution of altitude and describes the morphological distribution of a surface [29]. In our particular case, it corresponded to the ulcer surface. The DEM was used as the basis for creating the orthomosaic that corrected deformations caused by the perspective and reliefs, allowing us to keep all the information of the final reconstruction such as area, perimeter, and depth. Figure 2e shows the final 3D reconstruction. The metric surface area measurements were used to analyze the evolution of the ulcers [29,30].

**Figure 3:**
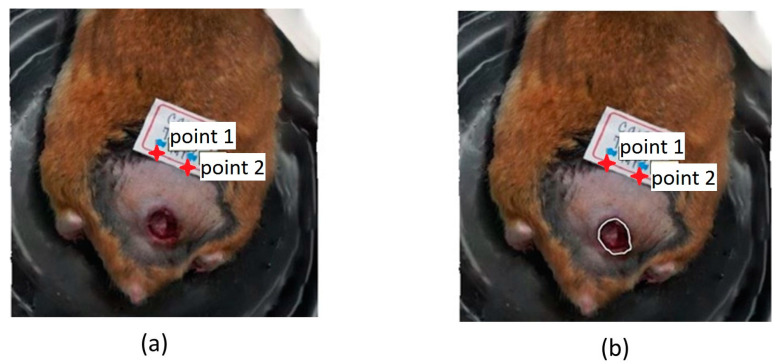
(**a**) Original 3D model; (**b**) Bordering the skin ulcer with the “draw polygon” tool (white polygon over the ulcer).

The area of the ulcer was measured as a criterion to determine the clinical improvement concerning the initial conditions [31], i.e., when the treatments started. Before the acquisition, calibration had to be performed in the software, positioning two points with a known separation between them, in our case 1 cm, and it was taken as a reference for area calculations. The measurement was obtained directly in the software using the “Draw Polygon” tool on the mentioned orthomosaic, bordering the lesion until obtaining a closed figure (Figure 3) from which the data corresponding to the area (m^2^) were extracted. To outline the ulcer, it was important to identify its center and the areas of the skin in which there was a significant change in color and texture.

### 2.4. Temporal Evaluation

The obtained metrics were recorded, distinguishing the hamster with the type of inoculated *Leishmania* and applied treatment. The data were used to graph the ulcer area with respect to the measurement acquisition date. This allowed us to observe the ulcer disease’s evolution and determine the ulcer’s reduction [32]. This process was carried out by grouping the hamsters in each graph according to the type of inoculated *Leishmania* species and applied treatment. A total of four graphs were obtained: LBCT, LBET, LPCT, and LPET.

### 2.5. Correlation between Optical Parameters and Surface Area

From the values of the optical parameters obtained previously [18], a correlation study was carried out with the metric parameter surface area. The optical parameters considered for this study are presented in Table 1. This correlation allowed us to determine the degree of association of the two variables quantitatively and to identify if their relationship was negative or positive and their degree of association. The interdependence between the two variables was quantified with the Rho correlation coefficient, assuming values equal to or greater than 0.7 as a moderate–strong to perfect relationship [33]. The correlation between the variables was made using the MATLAB program. The optical parameters were obtained from an inverse modeling procedure applied to diffuse reflectance spectra acquired from the golden hamsters’ skin ulcers. The spectra were obtained using a commercial spectrometer. Then, they were calibrated using black and white reference patterns. The calibrated spectra were processed using an inverse modeling procedure. This procedure consisted of a direct model, in which simulated reflectance was calculated by evaluating different values for the 10 previously mentioned optical parameters in an exponential light–tissue interaction equation. Then, the simulated reflectance was approximated to a measured one using an optimization approach. This was carried out by adjusting the 10 optical parameters until a minimum mean squared error between the calculated and the measured reflectance was achieved [18].

## 3. Results

### 3.1. Surface Area Evolution 

**Figure 4:**
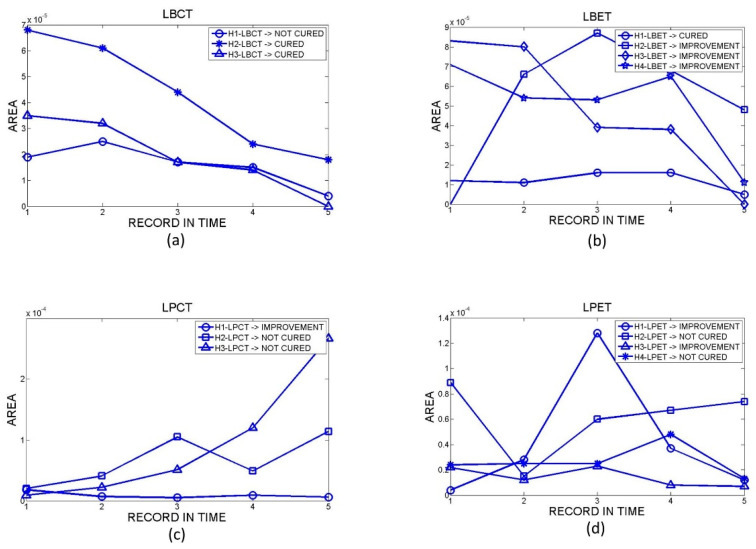
Temporal evolution of the surface area of skin ulcers in hamsters with (**a**) LBCT, (**b**) LBET, (**c**) LPCT, and (**d**) LPET.

Only 14 hamsters arrived at the end of the post-treatment period (3 LBCT, 4 LBET, 3 LPCT, and 4 LPET). A total of 70 orthomosaics were obtained from the 14 hamsters, with five measurement dates for each one. Figure 4 shows the results obtained for the temporal evolution of skin ulcers’ surface areas. In the figure, the values of the ulcers’ surface areas (in m^2^-Y axis) are plotted against the data collection dates (X axis).

Table 2 shows the values of the ulcer’s surface areas obtained from the software.

Hamsters identified as LBCT denoted a tendency to decrease their ulcer area over time. Figure 4a shows that the most significant area reduction began at the end of the treatment after the second measurement. The area was reduced almost entirely for hamsters identified as H2-LBCT and H3-LBCT. These hamsters were classified as cured according to the clinical report. In the case of the hamster identified as H1-LBCT, their clinical report classed them as not cured.

Hamsters identified as LBET tended to decrease their ulcer area significantly after the fourth measurement date with an almost total reduction. According to the clinical report, hamsters H2-LBET, H3-LBET, and H4-LBET were classified as improved, and H1-LBET as cured.

For the group of hamsters inoculated with *L. braziliensis*, there was an improvement in the injured area for both types of treatments. However, for hamsters treated with LBCT, faster and more constant action was evidenced over time, unlike hamsters treated with LBET, which experienced a faster effect but with a late onset.

Hamsters identified as LPCT showed constant variation in the area of the lesion without evident improvement during the study. They were classified according to clinical reports as follows: H1-LPCT as improvement and H2-LPCT and H3-LPCT as not cured.

Likewise, for hamsters identified as LPET, their data also presented variations, and they were classified as follows: H1-LPET and H3-LPET as improvement and H2-LPET and H4-LPET as not cured.

For the group of hamsters inoculated with *L. panamensis*, there was no clear trend in the evolution of the ulcer size.

**Figure 5:**
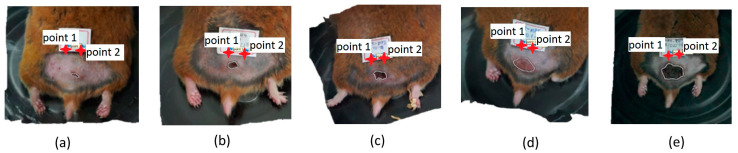
Evolution of the hamster with LPCT identified as H3-LPCT. (**a**) First, (**b**) second, (**c**) third, (**d**) fourth, and (**e**) fifth measurement date. Bordering the skin ulcer with the “draw polygon” tool produces the white polygon over the ulcer.

An important factor for the variation in the area measurements during the study is that not only was the ulcer analyzed, but also the area of injured skin. Physicians identify injured skin by analyzing visual changes in color and texture in the patient’s skin. Thus, after the ulcer has healed, the injured skin area that will appear in its place could be more significant. It showed an increase in the area when a reduction was expected. An ulceration phase can also start from this injured area again, as in the H3-LPCT hamster (Figure 5).

These manual measurements, which corresponded to the clinical criterion, depended on a visual inspection; therefore, this measurement was subjective to the observer. So, an ulcer that had a small area, to the observer, may have been a healed lesion with a surface area of zero, hence the importance of having software such as that proposed in this article to reduce the subjectivity of measurement.

### 3.2. Correlation Area vs. Optical Values

The disease’s evolution generally presented different responses in each hamster that was followed during the study. Thus, the different phases of wound healing and their duration, the immune response that each animal presents, and the biological agents involved in the process will be decisive for interpreting the existing relationship between optical parameters and area surface.

The results of the correlation between the optical parameters (#OP) and the ulcer area for each hamster are shown in Table 3. The *p* values around 0.1 are highlighted in bold letters.

**Figure 6:**
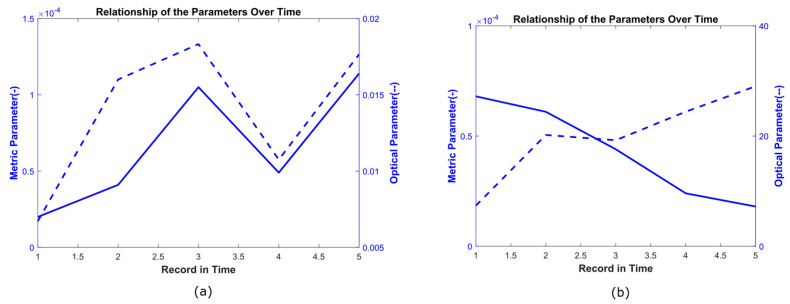
Direct and inverse relationship between the optical parameter (dashed line) and the metric parameter (continuous line). (**a**) Direct relationship between area and epidermis for the hamster identified as H3-LBET. (**b**) Inverse relationship between area and keratinocytes for the hamster identified as H2-LBCT.

The relationship between both parameters could be direct (Figure 6a) when the correlation coefficient was positive, indicating that when the area parameter increased, the optical parameter also increased. Alternatively, the relationship could be inverse (Figure 6b) when the correlation coefficient was negative, indicating opposite behavior between both parameters. That is, when the area parameter increased, the optical parameter decreased. These relationships were interpreted according to the physiological characteristics of each hamster and the phase of the healing process. An analysis of these relationships is presented in the discussion section.

## 4. Discussion

Concerning the correlation between area and optical parameters, the obtained results show the following relationships:

### 4.1. Epidermis Thickness

The epidermis thickness optical parameter and the ulcer’s area showed a direct relationship for hamsters identified as H1-LBCT (not cured) and H2-LBCT (cured), H2-LBET and H3-LBET (both improved), and H2-LPCT (not cured). Since the epidermis is the first layer of the skin, the inverse behavior would be expected, where the greater the area of injury, the lower the value of the epidermis. However, the inverse behavior was evident and can be explained by the presence of a scab in the area of the wound, which are temporary skin formations that cover a healing wound and serve as a protective barrier for the regenerating dermis [34].

### 4.2. Collagen Diameter and VF Collagen

The ratio of the area to the diameter of collagen depends strictly on the duration of the ulceration and healing phases and the wound’s physical and morphological characteristics.

These aspects are the ones that mainly determine the distribution of collagen fibers and how their grouping occurs since a disorderly grouping will contribute to the greater or lesser diameter of the fibers and the aesthetic appearance of the scar [35]. This behavior is evidenced by the inverse relationship between the parameters for hamsters H3-LBCT (cured), and H4-LBET (improvement), which presented granulomas and clefts in their healing, respectively. For the hamsters identified as H2-LBCT (cured), H2-LBET (cured), and H3-LPCT (not cured), the relationship between the variables was direct. In these hamsters, the healing process was more efficient, and the scars had a better physical appearance.

This same fact explains the relationship between collagen’s optical parameter VF and the area. A direct relationship was evident in the hamster identified as H2-LBCT (cured). An inverse relationship was identified for H1-LBCT (not cured) and H3-LPET (improvement).

### 4.3. Keratinocytes

Concerning the keratinocytes optical parameter and the area of the lesion, ulcers in hamsters H2-LBCT (cured) and H3-LBET (improvement) were both in the healing phase, where keratinocytes have lower proliferation and are responsible for the production of keratin, one of the main components of the skin [36]. Therefore, the larger the area of the lesion, the lower the proportion of keratinocytes. Keratin will present high values once the tissue is wholly regenerated.

Contrary to this, the behavior of the hamster identified as H1-LPET was classified as improvement. The proportion of keratinocytes was directly proportional to the ulcer’s size, which was not in the healing phase but in defense against an active infection by the *Leishmania* parasite. Therefore, keratin would have been present during the resolution of the infection, re-establishing itself at the epidermis level.

### 4.4. Oxygen Saturation and VF Blood 

The relationship between the SpO_2_ optical parameter and the injury area was direct. This was due to the beginning of the proliferation phase where angiogenesis occurs to irrigate the ulcer to provide oxygen and essential nutrients that contribute to the generation of new epithelial tissue. Therefore, it was expected that the greater the area of injury, the greater the angiogenesis process and the higher the oxygen levels in the lesion area. This behavior was evident in the hamsters identified as H3-LBET (improvement) and H4-LPET (not cured).

On the other hand, hamster H2-LBCT (cured) presented contrary behavior. For this hamster, the healing process was due to fibrosis, characterized by a lack in adequate vascularization and little irrigation of nutrients and O_2_ in the injury area.

The same explanation was given for the optical parameter volume fraction of blood. The amount of blood irrigated strictly depended on the angiogenesis process, with a direct relationship for hamsters identified as H1-LPET (improvement) and H3-LPET (improvement). For the hamster identified as H2-LBET (improvement), the opposite relationship between optical and area parameters was caused by the tissue’s current fibrosis and the poor vascularization process.

From a metrological point of view, the validation of the technique’s accuracy by measuring an object with a known size has been already presented in the literature by other authors [10,37,38]. However, the presented method needs further investigation, and indeed, the accuracy must be validated for the full 3D reconstruction of the topography of an ulcer. The conventional procedure for wound healing assessment is based on a manual ruler method (a non-3D method), which is suggested in many medical wound care guidelines and specialized communities [6,7]. Some initial results of comparing measurements of surface area 3D reconstruction against the non-3D CL clinical conventional method were presented in [15].

## 5. Conclusions

The area is the fundamental criterion for the clinical follow-up of cutaneous ulcers caused by Leishmaniasis. This measurement is carried out by healthcare personnel with basic measuring instruments. Since these are observer-dependent analyses, it is necessary to develop new control strategies that allow us to make decisions concerning the treatment and determine if it is effective or if it requires any modifications. In this article, we proposed the use of 3D reconstructions together with optical values as a tool to assist the follow-up of treated CL ulcers. This study was conducted in golden hamsters inoculated with two species of *leishmanias*: *L. braziliensis* and *L. panamensis*. The hamsters were treated either with a commercial or an experimental medicament.

*L. panamensis* does not show an explicit response to treatment in 45 days. The tendency is ill-defined for both types of treatments, unlike *L. braziliensis,* where the tendency is to reduce the area for both types of treatment.

Some of the optical parameters correlated with the ulcer’s surface area. Those optical parameters that did show a close correlation with the surface area were epidermis thickness, collagen diameter, volume fraction of collagen, keratinocytes, oxygen saturation, and FV blood. These correlations show that optical tools could support the analysis of area measurements. This study is a proof of concept that provides the starting point for future studies in which, with a larger sample size, the possibility of predicting the evolution of leishmaniasis could be analyzed to give a more reliable concept of cures.

Future work will seek to carry out a comparison with other 3D surface reconstruction techniques in order to corroborate the accuracy of the used system. Also, temporal measurements involving 3D surface measurements, optical, and histopathological analysis could also be performed in order to correlate the changes in both metrological and biological parameters.

## Figures and Tables

**Table 1:** Optical parameters considered for this study.

#Optical Parameter (#OP)	Optical Parameter Name
1	Epidermis thickness
2	Epidermis + dermis thickness
3	Diameter of keratinocytes
4	Diameter of collagen
5	Volume fraction (VF) of collagen
6	Diameter of fibroblast
7	Diameter of macrophages
8	VF of melanin
9	VF of blood
10	Oxygen saturation

**Table 2:** Ulcer area (m^2^) during the five chosen measurement dates obtained from the software.

Ulcer Area on the Five Measurement Dates (m^2^)					
	Date	LBCT	LBET	LPCT	LPET
		Area	Area	Area	Area
H1	1	0.19	0.12	0.18	0.04
	2	0.25	0.11	0.07	0.28
	3	0.17	0.16	0.05	1.28
	4	0.15	0.16	0.09	0.37
	5	0.04	0.05	0.06	0.12
H2	1	0.68	0.00	0.20	0.89
	2	0.61	0.66	0.41	0.15
	3	0.44	0.87	1.05	0.60
	4	0.24	0.68	0.49	0.67
	5	0.18	0.48	1.14	0.74
H3	1	0.35	0.83	0.09	0.22
	2	0.32	0.80	0.22	0.12
	3	0.17	0.39	0.51	0.23
	4	0.14	0.38	1.20	0.08
	5	0.00	0.00	2.67	0.07
H4	1		0.71		0.24
	2		0.54		0.25
	3		0.53		0.25
	4		0.65		0.48
	5		0.11		0.13

**Table 3:** ANOVA analysis of the correlation between optical parameters and area. The optical parameters are 1. epidermis thickness, 2. epidermis + dermis thickness, 3. diameter of keratinocytes, 4. diameter of collagen, 5. volume fraction of collagen, 6. diameter of fibroblast, 7. diameter of macrophages, 8. volume fraction of melanin, 9. volume fraction of blood, and 10. oxygen saturation. Correlations with a Rho-value greater than 0.7 and a *p*-value (*p*) less than 0.2 are highlighted.

Correlation between Optical Parameters and Area															
		Hamster													
#OP	ANOVA	LBCT			LBET				LPCT			LPET			
		H1	H2	H3	H1	H2	H3	H4	H1	H2	H3	H1	H2	H3	H4
1	Rho	***0.70***	***0.95***	0.43	−0.17	***0.75***	***0.75***	0.47	0.44	***0.83***	−0.44	0.42	−0.09	0.62	−0.11
	*p*	***0.19***	***0.01***	0.47	0.79	***0.15***	***0.14***	0.42	0.46	***0.08***	0.46	0.49	0.88	0.27	0.86
2	Rho	−0.38	***0.84***	0.51	−0.49	0.60	−0.22	0.31	0.11	***0.85***	−0.39	−0.42	0.07	−0.51	0.00
	*p*	0.53	***0.07***	0.38	0.41	0.29	0.72	0.61	0.86	***0.07***	0.51	0.48	0.90	0.38	1.00
3	Rho	−0.36	***−0.88***	−0.17	0.21	−0.35	***−0.75***	0.25	−0.01	0.54	0.14	***0.89***	0.08	−0.39	−0.22
	*p*	0.55	***0.05***	0.78	0.74	0.56	***0.14***	0.68	0.99	0.34	0.82	***0.04***	0.90	0.51	0.72
4	Rho	−0.22	***0.90***	***−0.90***	***0.85***	−0.32	0.48	***−0.78***	0.04	−0.06	***0.86***	0.63	−0.57	0.61	−0.64
	*p*	0.73	***0.04***	***0.03***	***0.07***	0.59	0.42	***0.12***	0.95	0.93	***0.06***	0.26	0.32	0.27	0.25
5	Rho	***−0.71***	***0.75***	0.09	−0.33	0.21	−0.37	0.41	−0.35	0.63	0.09	−0.32	−0.19	***−0.96***	0.16
	*p*	***0.18***	***0.14***	0.88	0.59	0.74	0.54	0.49	0.56	0.26	0.89	0.60	0.76	***0.01***	0.80
6	Rho	−0.66	***−0.74***	−0.42	0.32	−0.45	−0.29	−0.42	−0.45	−0.67	0.26	0.35	0.20	***0.87***	−0.17
	*p*	0.23	***0.15***	0.49	0.60	0.45	0.63	0.48	0.44	0.21	0.67	0.57	0.74	***0.06***	0.79
7	Rho	0.47	−0.39	−0.24	−0.02	***−0.87***	0.19	−0.43	0.38	−0.51	0.28	0.02	0.11	0.26	−0.30
	*p*	0.42	0.52	0.70	0.97	***0.06***	0.76	0.47	0.52	0.38	0.64	0.98	0.86	0.67	0.63
8	Rho	−0.46	−0.50	−0.24	0.17	−0.67	−0.13	0.11	−0.10	−0.59	0.04	0.47	0.19	0.12	0.50
	*p*	0.43	0.39	0.70	0.78	0.22	0.84	0.87	0.88	0.29	0.95	0.43	0.76	0.85	0.39
9	Rho	−0.09	0.62	0.08	0.21	***−0.79***	0.24	−0.61	0.55	0.00	−0.40	***0.77***	−0.38	***0.88***	−0.24
	*p*	0.88	0.26	0.89	0.73	***0.11***	0.70	0.28	0.34	1.00	0.51	***0.13***	0.52	***0.05***	0.70
10	Rho	0.58	***−0.84***	−0.43	−0.21	−0.61	***0.81***	−0.49	−0.26	0.14	0.56	0.26	−0.04	0.09	***0.85***
	*p*	0.30	***0.08***	0.47	0.74	0.27	***0.09***	0.41	0.67	0.82	0.33	0.67	0.95	0.89	***0.07***

## Data Availability

Data are unavailable due to privacy.

## References

[1] (2020). World Health Organization: Leishmaniasis. www.who.int/es/news-room/fact-sheets/detail/leishmaniasis.

[2] Vieira V.R., de Aguiar G.M., de Azevedo A.C.R., Rangel E.F., Guimarães A.É. (2023). Sandfly population dynamics in areas of American cutaneous leishmaniasis, Municipality of Paraty, Rio de Janeiro, Brazil. Sci. Rep..

[3] Suprien C., Rocha P.N., Teixeira M., Carvalho L.P., Guimarães L.H., Bonvoisin T., Machado P.R.L., Carvalho E.M. (2020). Clinical Presentation and Response to Therapy in Children with Cutaneous Leishmaniasis. Am. J. Trop. Med. Hyg..

[4] López-Carvajal L., Vélez I., Arbeláez M.P., Olliaro P. (2018). Eligibility criteria and outcome measures adopted in clinical trials of treatments of cutaneous leishmaniasis: Systematic literature review covering the period 1991–2015. Trop. Med. Int. Health.

[5] Cota G.F., de Sousa M.R., Fereguetti T.O., Saleme P.S., Alvarisa T.K., Rabello A. (2016). The Cure Rate after Placebo or No Therapy in American Cutaneous Leishmaniasis: A Systematic Review and Meta-Analysis. PLoS ONE.

[6] Haesler E., European Pressure Ulcer Advisory Panel, National Pressure Injury Advisory Panel and Pan Pacific Pressure Injury Alliance (2019). Prevention and Treatment of Pressure Ulcers/Injuries: Clinical Practice Guideline.

[7] Skilled Wound Care How to Measure Wounds, the Right Way. https://www.skilledwoundcare.com/post/how-to-measure-wounds-the-right-way#:~:text=The%20wound%20is%20typically%20measured,with%20the%20tip%20of%20finger.

[8] Garzón-Márquez C., Gómez-Ramírez M., Murillo J.D., Robledo S., Hernandez A., Castañeda B., Pérez-Buitrago S. Follow-up of Cutaneous Leishmaniasis by Three-Dimensional Reconstruction Based on Photogrammetry: Proof of Concept. Proceedings of the VIII Latin American Conference on Biomedical Engineering and XLII National Conference on Biomedical Engineering: Proceedings of CLAIB-CNIB 2019.

[9] Lucas Y., Niri R., Treuillet S., Douzi H., Castaneda B. (2021). Wound Size Imaging: Ready for Smart Assessment and Monitoring. Adv. Wound Care.

[10] Zenteno O., González E., Treuillet S., Valencia B.M., Castaneda B., Llanos-Cuentas A., Lucas Y. (2019). Volumetric monitoring of cutaneous leishmaniasis ulcers: Can camera be as accurate as laser scanner?. Comput. Methods Biomech. Biomed. Eng. Imaging Vis..

[11] Filko D., Nyarko E.K. (2023). 2D/3D Wound Segmentation and Measurement Based on a Robot-Driven Reconstruction System. Sensors.

[12] Anisuzzaman D.M., Wang C., Rostami B., Gopalakrishnan S., Niezgoda J., Yu Z. (2022). Image-Based Artificial Intelligence in Wound Assessment: A Systematic Review. Adv. Wound Care.

[13] Casas L., Treuillet S., Valencia B.M., Llanos A., Castaneda B. Low-cost uncalibrated video-based tool for tridimensional reconstruction oriented to assessment of chronic wounds. Proceedings of the 10th International Symposium on Medical Information Processing and Analysis.

[14] Stark E., Haffner O., Kučera E. (2022). Low-Cost Method for 3D Body Measurement Based on Photogrammetry Using Smartphone. Electronics.

[15] Viloria C., Londoño S., Murillo J., Perez S., Galeano J., Zarzycki A., Garzón J., Robledo S.M. (2022). Comparison between clinical and computational method of surface measurements of skin ulcers caused by Cutaneous Leishmaniasis. Opt. Pura Apl..

[16] Nouri D., Lucas Y., Treuillet S., Jolivot R., Marzani F. (2013). Colour and multispectral imaging for wound healing evaluation in the context of a comparative preclinical study. Medical Imaging 2013: Image Processing.

[17] Squiers J.J., Thatcher J.E., Bastawros D.S., Applewhite A.J., Baxter R.D., Yi F., Quan P., Yu S., DiMaio J.M., Gable D.R. (2021). Machine learning analysis of multispectral imaging and clinical risk factors to predict amputation wound healing. J. Vasc. Surg..

[18] Botina D., Franco R., Murillo J., Galeano J., Zarzycki A., Torres-Madronero M.C., Bermúdez C., Montaño J., Garzón J., Marzani F. (2019). Estimation of Biological Parameters of Cutaneous Ulcers Caused by Leishmaniasis in an Animal Model Using Diffuse Reflectance Spectroscopy. Sensors.

[19] Restrepo L., Murillo J., Botina D., Zarzycki A., Garzón J., Franco R., Montano J., Calderon S., Torres-Madronero M.C., Marzani F. (2021). Diffuse Reflectance Parameters of Treated Leishmaniasis Cutaneous Ulcers and Association with Histopathologies in an Animal Model: A Proof of Concept. SLAS Technol. Transl. Life Sci. Innov..

[20] Yudovsky D., Nouvong A., Schomacker K., Pilon L. (2011). Monitoring temporal development and healing of diabetic foot ulceration using hyperspectral imaging. J. Biophotonics.

[21] Ewerlöf M., Strömberg T., Larsson M., Salerud E.G. (2022). Multispectral snapshot imaging of skin microcirculatory hemoglobin oxygen saturation using artificial neural networks trained on in vivo data. J. Biomed. Opt..

[22] Robledo S.M., Carrillo L.M., Daza A., Restrepo A.M., Muñoz D.L., Tobón J., Murillo J.D., López A., Ríos C., Mesa C.V. (2012). Cutaneous Leishmaniasis in the dorsal skin of hamsters: A useful model for the screening of antileishmanial drugs. J. Vis. Exp..

[23] Congreso de Colombia (1989). Ley 84 de 1989. “Estatuto Nacional de Protección de los Animales”.

[24] Agisoft Agisoft Metashape User Manual Professional Edition, Version 1.5. https://www.agisoft.com/pdf/metashape-pro_1_5_en.pdf.

[25] Grandón-Pastén N., Aracena-Pizarro D., Tozzi C.L. (2007). Reconstrucción De Objeto 3D a Partir De Imágenes Calibradas. Ingeniare Rev. Chil. Ing..

[26] Martínez J., Pérez-Palau D., Cilla M., Garrido N., Larrañaga A., Pérez-Rey I. (2023). Semi-Automatic 3D Reconstruction of Atheroma Plaques from Intravascular Ultrasound Images Using an ad-hoc Algorithm. Mathematics.

[27] Nabil M., Saleh F. 3D reconstruction from images for museum artefacts: A comparative study. Proceedings of the 2014 International Conference on Virtual Systems & Multimedia (VSMM).

[28] Savage J., Jeffery S. (2013). Use of 3D photography in complex-wound assessment. J. Wound Care.

[29] Aravena P.C., Sandoval S.P., Pizarro F.E., Simpson M.I., Castro-Adams N., Serandour G., Rosas C. (2020). Leukocyte and Platelet-Rich Fibrin Have Same Effect as Blood Clot in the 3-Dimensional Alveolar Ridge Preservation. A Split-Mouth Randomized Clinical Trial. J. Oral Maxillofac. Surg..

[30] Wechsler S.P. Digital Elevation Model (DEM) Uncertainty: Evaluation and Effect on Topographic Parameters. Proceedings of the ESRI User Conference.

[31] Schuster S., Hartley M.-A., Tacchini-Cottier F., Ronet C. (2014). A scoring method to standardize lesion monitoring following intra-dermal infection of Leishmania parasites in the murine ear. Front. Cell. Infect. Microbiol..

[32] Arevalo I., Tulliano G., Quispe A., Spaeth G., Matlashewski G., Llanos-Cuentas A., Pollack H. (2007). Role of Imiquimod and Parenteral Meglumine Antimoniate in the Initial Treatment of Cutaneous Leishmaniasis. Clin. Infect. Dis..

[33] Hauke J., Kossowski T. (2011). Comparison of Values of Pearson’s and Spearman’s Correlation Coefficients on the Same Sets of Data. Quaest. Geogr..

[34] González K., Diaz R., Ferreira A.F., García V., Paz H., Calzada J.E., Ruíz M., Laurenti M., Saldaña A. (2018). Histopathological characteristics of cutaneous lesions caused by Leishmania Viannia panamensis in Panama. Revista do Instituto de Medicina Tropical de São Paulo.

[35] Gauglitz G.G., Jeschke M., Kamolz L.P., Shahrokhi S. (2013). Wound Healing and Wound Car. Burn Care and Treatment.

[36] Nylén S., Eidsmo L. (2012). Tissue damage and immunity in cutaneous leishmaniasis. Parasite Immunol..

[37] Li X., Chen Z., Zhang L., Jia D. (2016). Construction and accuracy test of a 3D model of non-metric camera images using Agisoft PhotoScan. Procedia Environ. Sci..

[38] Ðuric I., Vasiljevic I., Obradovic M., Stojakovic V., Kicanovic J., Obradovic R. Comparative Analysis of Open-Source and Commercial Photogrammetry Software for Cultural Heritage. Proceedings of the eCAADe 2021 International Scientific Conference.

